# GRSF1 Regulates RNA Processing in Mitochondrial RNA Granules

**DOI:** 10.1016/j.cmet.2013.02.005

**Published:** 2013-03-05

**Authors:** Alexis A. Jourdain, Mirko Koppen, Mateusz Wydro, Chris D. Rodley, Robert N. Lightowlers, Zofia M. Chrzanowska-Lightowlers, Jean-Claude Martinou

**Affiliations:** 1 Department of Cell Biology, University of Geneva, 30 Quai Ernest-Ansermet, 1211 Genève 4, Switzerland; 2 Wellcome Trust Centre for Mitochondrial Research, Institute for Ageing and Health, Newcastle University, Medical School, Framlington Place, Newcastle upon Tyne NE2 4HH, UK

## Abstract

Various specialized domains have been described in the cytosol and the nucleus; however, little is known about compartmentalization within the mitochondrial matrix. GRSF1 (G-rich sequence factor 1) is an RNA binding protein that was previously reported to localize in the cytosol. We found that an isoform of GRSF1 accumulates in discrete foci in the mitochondrial matrix. These foci are composed of nascent mitochondrial RNA and also contain RNase P, an enzyme that participates in mitochondrial RNA processing. GRSF1 was found to interact with RNase P and to be required for processing of both classical and tRNA-less RNA precursors. In its absence, cleavage of primary RNA transcripts is abnormal, leading to decreased expression of mitochondrially encoded proteins and mitochondrial dysfunction. Our findings suggest that the foci containing GRSF1 and RNase P correspond to sites where primary RNA transcripts converge to be processed. We have termed these large ribonucleoprotein structures “mitochondrial RNA granules.”

## Graphical abstract

**Graphical Abstract:**

**Figure:**
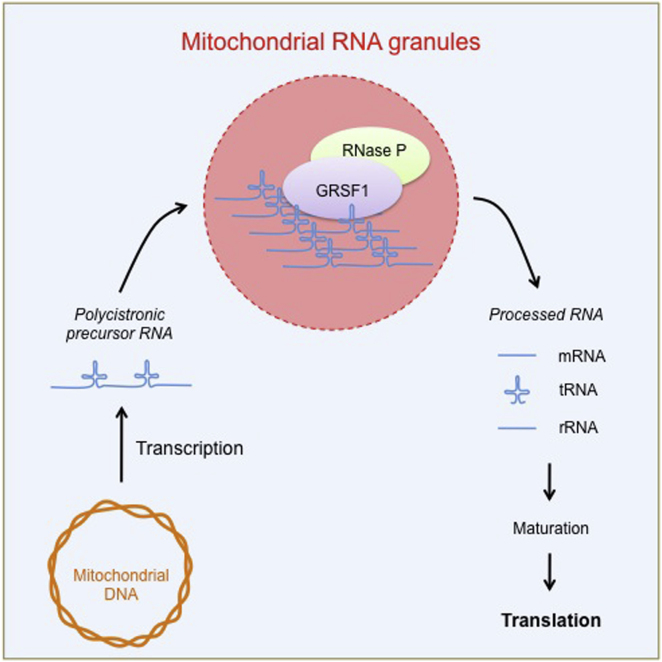

**Highlights:**

► GRSF1 resides in the mitochondrial matrix and is required for mitochondrial function ► GRSF1 is required for the processing of tRNA-containing and tRNA-lacking precursors ► GRSF1, RNase P, and nascent RNA are part of “mitochondrial RNA granules” ► Mitochondrial RNA granules are functionally linked to RNA processing

## Introduction

Mitochondria are involved in many metabolic processes, a primary function being to convert energy from nutrients into ATP through oxidative phosphorylation. These organelles are thought to derive from an ancient endosymbiotic event between an α-proteobacterium and the ancestor of modern eukaryotic cells. Over evolutionary time most of the ancestral bacterial genes have been transferred to the nucleus, requiring >1,000 nuclear-encoded proteins to translocate to the mitochondrion following cytosolic synthesis (Pagliarini et al., 2008). As a result, most mitochondrial genomes are generally smaller than their bacterial ancestors, as is the case for the 16.6 kb human mitochondrial DNA (mtDNA).

The human mitochondrial genome encodes two ribosomal RNAs, 22 tRNAs, and 13 open reading frames (ORFs) that are transcribed and translated within the organelle. The mitochondrial genetic information is indispensable for the function of respiratory complexes I, III, IV, and V, as exemplified by the large number of human disorders attributed to mtDNA mutations and the role such mutations may play in aging (Trifunovic et al., 2004; Wallace, 1997).

Mitochondrial transcription occurs on both DNA strands and gives rise to three polycistronic primary transcripts (Montoya et al., 1983). The primary mitochondrial transcripts are composed of ORFs and rRNAs that are usually punctuated by tRNAs (Ojala et al., 1981). Cleavage of tRNAs at their 5′ and 3′ ends by RNase P and RNase Z, respectively, releases individual RNA species from the polycistronic precursor that are further matured, as reviewed by Temperley et al. (2010). Defects in mitochondrial RNA (mtRNA) processing have been associated with several mitochondrial disorders (Suzuki et al., 2011), making it important to understand in more detail how mtRNA processing is spatially and temporally organized with regard to both transcription and translation.

Recently, one of the RNA processing enzymes was identified in mitochondria (Holzmann et al., 2008). It is a protein-only RNase P, composed of three subunits, MRPP1 (RG9MTD1), MRPP2 (HSD17B1), and MRPP3 (KIAA0391). These three proteins are necessary and sufficient for 5′ tRNA processing in vitro. An additional RNA-containing RNase P has been reported, but its substrate specificity has not been confirmed (Chen et al., 2006). Processing of the tRNA 3′ end is performed by an RNase Z type enzyme, namely ELAC2 (Brzezniak et al., 2011; Sanchez et al., 2011). Four of the mtORFs are not flanked on both sides by a tRNA. The 5′ junction of *MTCO1* with noncoding RNA (ncRNA) does not have an intervening canonical tRNA. However, the upstream ncRNA sequence has been proposed to adopt a tRNA-like structure, and so the processing of this transcription unit also depends on RNase P (Mercer et al., 2011; Sanchez et al., 2011). The three other ORF junctions that are devoid of tRNAs are *MTND6*-ncRNA, *MTND5*-*MTCYB*, and RNA14-*MTCO3*, and these do not depend on RNase P/Z for their processing (Brzezniak et al., 2011; Sanchez et al., 2011). In the case of *MTND5*-*MTCYB*, it has been shown that the pentatricopeptide-containing protein PTCD2 is necessary for processing (Xu et al., 2008), but, to our knowledge, no factor has been identified that can resolve the processing of the two others.

GRSF1 (G-rich sequence factor 1) was originally identified as a cytosolic RNA-binding protein with high affinity for G-rich sequences (Qian and Wilusz, 1994). GRSF1 belongs to the family of heterogeneous nuclear ribonucleoproteins F/H (hnRNP) (Schaub et al., 2007) and contains three quasi-RNA-recognition motifs (qRRM) (Ufer, 2012). Members of this family are involved in different steps of posttranscriptional RNA processing, such as RNA splicing, polyadenylation, and transport in RNA granules, as shown in oligodendrocytes (Han et al., 2010; White et al., 2012). Moreover, GRSF1 has been reported to play a role in the translation of mRNA species of both cellular and viral origin, including those containing IRES elements (Jablonski and Caputi, 2009; Kash et al., 2002; Park et al., 1999; Ufer et al., 2008). Thus, GRSF1 appears to be a multifunctional protein, whose function is likely to involve binding to G-rich sequences (Ufer, 2012). Recently, GRSF1 has been identified in a proteomic analysis of mitochondria and appeared in a list of proteins required for oxidative phosphorylation (Bayona-Bafaluy et al., 2011; R.N.L., unpublished data; Pagliarini et al., 2008). The mitochondrial function of GRSF1 remains to be elucidated.

Here, we report that an isoform of GRSF1 resides in a novel mitochondrial structure that we have called “mitochondrial RNA granules,” where it plays an essential role in precursor RNA processing.

## Results

### GRSF1 Is a Mitochondrial Protein

**Figure 1:**
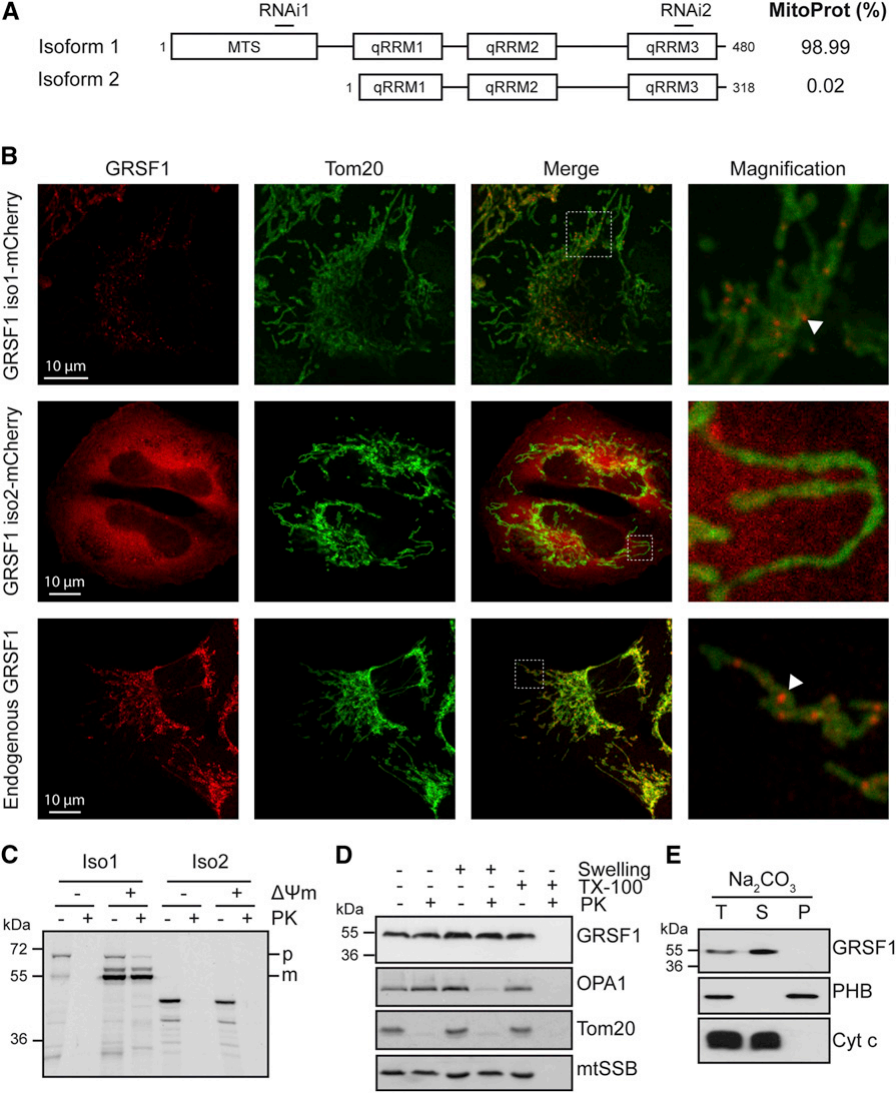
GRSF1 Is a Mitochondrial Protein (A) Schematic representation of GRSF1 isoform 1 and 2. MTS, mitochondrial targeting signal predicted by the MitoProt algorithm (http://ihg.gsf.de/ihg/mitoprot.html). qRRM, quasi-RNA-recognition motif. RNAi, targeting sites of GRSF1 RNAi1 and RNAi2. MitoProt, probability of mitochondrial import according to MitoProt algorithm. (B) Confocal analysis of GRSF1 isoforms. HeLa cells were transfected with GRSF1-isoform1-mCherry (top panel) or GRSF1-isoform2-mCherry (middle panel) and immunolabeled with anti-Tom20. Bottom panel, endogenous GRSF1 and Tom20 were immunolabeled in 143B cells. Arrowheads, foci formed by GRSF1 in mitochondria. (C) Import of radiolabeled in vitro-translated GRSF1 isoforms into isolated 143B mitochondria. p, precursor. m, mature. ΔΨm, mitochondrial membrane potential. PK, Proteinase K. Note that precursor GRSF1 migrates at ∼70 kDa instead of the predicted 53 kDa, and mature GRSF1 runs at ∼55 kDa instead of the predicted 43 kDa. (D) Immunoblot analysis of mitochondrial extract after Proteinase K accessibility test. PK, Proteinase K. TX-100, Triton X-100. OPA1 is a mitochondrial intermembrane space protein anchored to the inner membrane; Tom20 is a mitochondrial outer-membrane protein; mtSSB is a mitochondrial matrix protein. Note that mature endogenous GRSF1 migrates at ca 55kDa, similar to in vitro-translated GRSF1 after import (C). (E) Immunoblot analysis of mitochondrial extract after alkaline sodium carbonate (Na_2_CO_3_) extraction. T, total extract. S, supernatant. P, pellet. PHB, prohibitin 1. Cyt c, cytochrome *c*.

We investigated whether several isoforms of GRSF1 might be responsible for the multiple roles attributed to the gene. According to the NCBI database, the *GRSF1* gene encodes two splice variants (i.e., isoform 1 [NM_002092] and isoform 2 [NM_001098477]; Figure 1A). The longer mRNA corresponds to isoform 1 and encodes a 480 amino acid protein. Isoform 2 contains an alternate exon in the 5′ coding region and utilizes a downstream start codon. Thus, the resulting isoform 2 has a shorter N terminus that lacks the first 162 amino acids present in isoform 1. The three conserved qRRM are present in both GRSF1 variants. Mitochondrial targeting prediction tools (MitoProt II [Claros and Vincens, 1996]) yield a probability of 98.99% that isoform 1 has a mitochondrial localization signal, with a cleavage site predicted after tyrosine 118. In contrast, isoform 2 is not predicted to have any mitochondrial localization signal (0.02%, MitoProt; Figure 1A).

We tagged both isoforms of GRSF1 at their carboxyl termini and expressed them in HeLa cells. GRSF1 isoform 1 was observed in mitochondria, where it colocalized with the mitochondrial protein Tom20 (Figure 1B). Intriguingly, this isoform was concentrated in discrete foci inside the mitochondrial tubules. In contrast, expression of isoform 2 resulted in a diffuse staining in both the cytosol and nucleus. The different localizations between GRSF1 isoforms were confirmed in mitochondrial import assays where we could successfully import radiolabeled in vitro-translated GRSF1 isoform 1, but not isoform 2, into mitochondria isolated from 143B cells (Figure 1C). This import was dependent on the membrane potential (ΔΨm), and after import we observed presequence cleavage of GRSF1 isoform 1, which was not further investigated.

**Figure 2:**
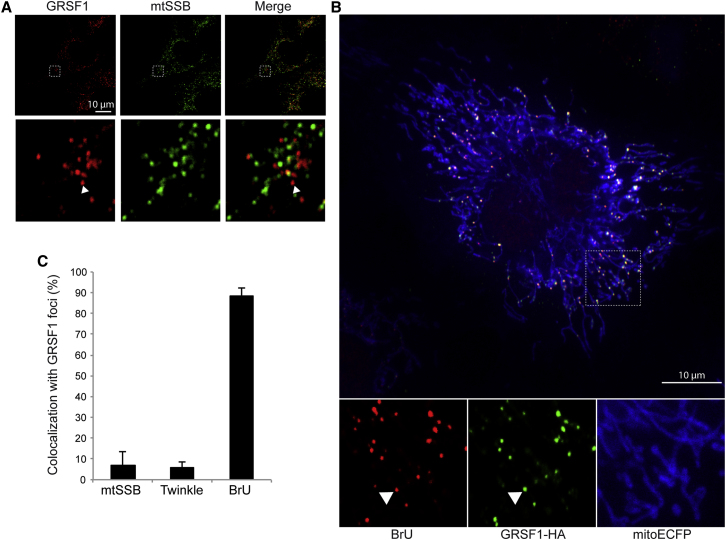
GRSF1 Colocalizes with Nascent Mitochondrial RNA (A) Confocal analysis of HeLa cells immunolabeled with anti-GRSF1 and anti-mtSSB. Arrowheads, foci formed by GRSF1 in mitochondria. Bottom panels, magnification. (B) Confocal analysis of HeLa cells transfected with GRSF1-HA, labeled with BrU, and immunostained with anti-HA and anti-BrU antibodies. Arrowheads, foci formed by GRSF1 in mitochondria. Bottom panels, magnification. (C) Quantification of (A) and (B). Data are shown ± SEM (N ≥ 5 cells with ≥20 foci per cell; in total ≥ 100 foci were analyzed).

To analyze the localization of endogenous GRSF1, 143B cells were immunolabeled with anti-GRSF1 and anti-Tom20 antibodies. GRSF1 was exclusively localized in mitochondria and accumulated in discrete foci in 143B or HeLa cells, suggesting that these cells preferentially express isoform 1 (Figure 1B and Figure 2A). Protein immunoblotting in different cell lysates confirmed the expression of isoform 1 in HeLa, 143B, HEK293T, and Jurkat cells (see Figure S1 online). In addition, in contrast to other cell types, Jurkat cells showed additional bands of lower molecular weight, which may correspond to additional isoforms. In agreement with our import experiment, we observed that endogenous GRSF1 isoform 1 was protected from degradation when mitochondria isolated from 143B cells were exposed to Proteinase K, even when mitochondria were swollen to rupture their outer membrane (Figure 1D). These data indicate a matrix localization of the protein, and alkaline sodium carbonate treatment demonstrated that it was not integrated in the inner mitochondrial membrane (Figure 1E). Thus, in diverse cell types, GRSF1 is a mitochondrial matrix protein that is organized in discrete foci.

### GRSF1 Colocalizes with Nascent Mitochondrial RNA in Mitochondrial RNA Granules

Next we characterized the nature of the foci formed by GRSF1. To assess whether the foci depend on genetic material of mitochondrial origin, we immunolabeled GRSF1 in 143B ρ^0^ cells, which lack mtDNA and therefore mtRNA. Although GRSF1 was still localized to mitochondria in these cells, the protein was not concentrated in foci but was rather diffuse in the matrix, indicating that the formation of GRSF1 foci requires the presence of a mitochondrial genome (Figure S2). The GRSF1 foci were reminiscent in terms of both size and distribution of those formed by mtDNA nucleoids. To test if these foci had overlapping contents, we immunolabeled HeLa cells with antibodies against both GRSF1 and the nucleoid marker mitochondrial single-stranded DNA binding protein (mtSSB). Fewer than 8% of the GRSF1 foci were found to colocalize with mtSSB foci (Figures 2A and 2C). Similar results were obtained with another nucleoid marker, Twinkle-EGFP (Figure 2C). Since GRSF1 is an RNA-binding protein, we hypothesized that the GRSF1 foci may contain RNA and thus performed bromouridine (BrU) labeling experiments to detect nascent mtRNAs, as previously described (Iborra et al., 2004). Eighty-eight percent of BrU-labeled foci colocalized with GRSF1 foci, strongly suggesting that GRSF1 associates with nascent RNA in mitochondria (Figures 2B and 2C). We have named these foci composed of nascent RNA and GRSF1 “mitochondrial RNA granules” (MRGs).

### GRSF1 Is Required to Maintain Mitochondrial Gene Expression

**Figure 3:**
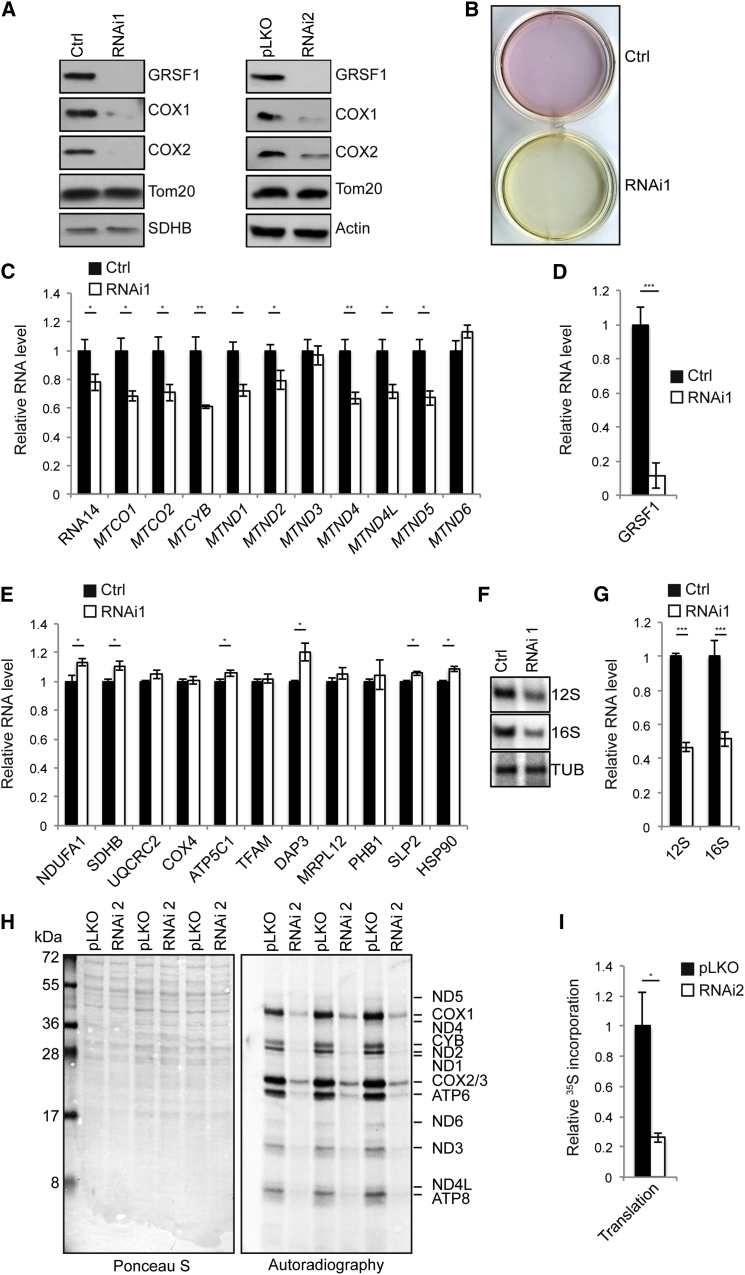
GRSF1 Is Required for Mitochondrial Gene Expression (A) Left panel, immunoblot analysis of 143B cells treated with control (Ctrl) or GRSF1 RNAi1. Right panel, immunoblot analysis of 143B cells infected with lentiviruses carrying an empty vector (pLKO) or a shRNA (RNAi2) against GRSF1. COX1 and COX2 are mitochondrially encoded proteins. SDHB and Tom20 are nuclear-encoded mitochondrial proteins. Actin is a nuclear-encoded cytosolic protein. (B) Acidification of the culture medium in GRSF1 RNAi1-treated 143B cells. The number of cells was the same in both conditions. Similar results were obtained in GRSF1 RNAi2-infected cells (data not shown). (C) NanoString analysis of 11 mitochondrially encoded ORFs in control (Ctrl) or GRSF1 RNAi1-treated 143B cells. RNA14 corresponds to bicistronic *MTATP8*-*MTATP6* RNA. Data are shown as mean ± SEM (n = 3). (D) NanoString analysis of GRSF1 in control (Ctrl) or GRSF1 RNAi1-treated 143B cells. Data are shown as mean ± SEM (n = 3). (E) NanoString analysis of 11 nuclear-encoded proteins in control (Ctrl) or GRSF1 RNAi1-treated 143B cells. Data are shown as mean ± SEM (n = 3). (F) Northern blot analysis of 12S and 16S rRNA in control (Ctrl) or GRSF1 RNAi1-treated cells. TUB, tubulin. (G) Phosphorimager quantification of (E). Data are shown as mean ± SEM (n = 3). (H) ^35^S-labeling of mitochondrial translation of 143B cells infected with lentiviruses carrying an empty vector (pLKO) or a shRNA (RNAi2) against GRSF1. (I) Phosphorimager quantification of (H). Data are normalized to the Ponceau S intensity and are shown as mean ± SEM (n = 4).

Given the colocalization of GRSF1 and nascent mtRNA, we tested whether GRSF1 may play a role in mtRNA metabolism. Downregulation of GRSF1 was obtained by lipotransfection of a synthetic RNAi (RNAi1) or by using lentiviral-mediated shRNA delivery (RNAi2) (Figure 1A and Figure 3A). Interestingly, we found that the protein levels of COX1 and COX2, two mitochondrially encoded proteins, were significantly reduced in both RNAi1-treated and RNAi2-expressing cells (Figure 3A). The effect of GRSF1 RNAi on mitochondrially encoded proteins was specific to the mitochondrial form of GRSF1, since RNAi1 only targets isoform 1 (Figure 1A). GRSF1-depleted cells were found to adopt a glycolytic metabolism, as shown by acidification of the culture medium, a common feature observed when cells cannot properly perform oxidative phosphorylation (Figure 3B). To further understand the role of GRSF1 on mitochondrial function, we analyzed the levels of mitochondrially encoded RNAs in GRSF1-depleted 143B cells and found that GRSF1 depletion affected 9 of the 11 mRNA transcription units tested (Figure 3C). As expected, GRSF1 isoform 1 transcripts themselves were reduced by >85% (Figure 3D), whereas nuclear-encoded transcripts were unaffected or slightly upregulated (Figure 3E). These results were confirmed by northern analysis of RNAi2-treated cells and also showed that rRNA and certain tRNA were affected by the absence of GRSF1 (Figures 3F and 3G and Figure 5). In accordance with these results, we observed a global decrease in mitochondrial translation products in RNAi2-infected 143B cells (Figures 3H and 3I). Together these results show that GRSF1 is required to maintain mitochondrial gene expression in 143B cells.

### RNase P Interacts and Colocalizes with GRSF1 in Mitochondrial RNA Granules

**Figure 4:**
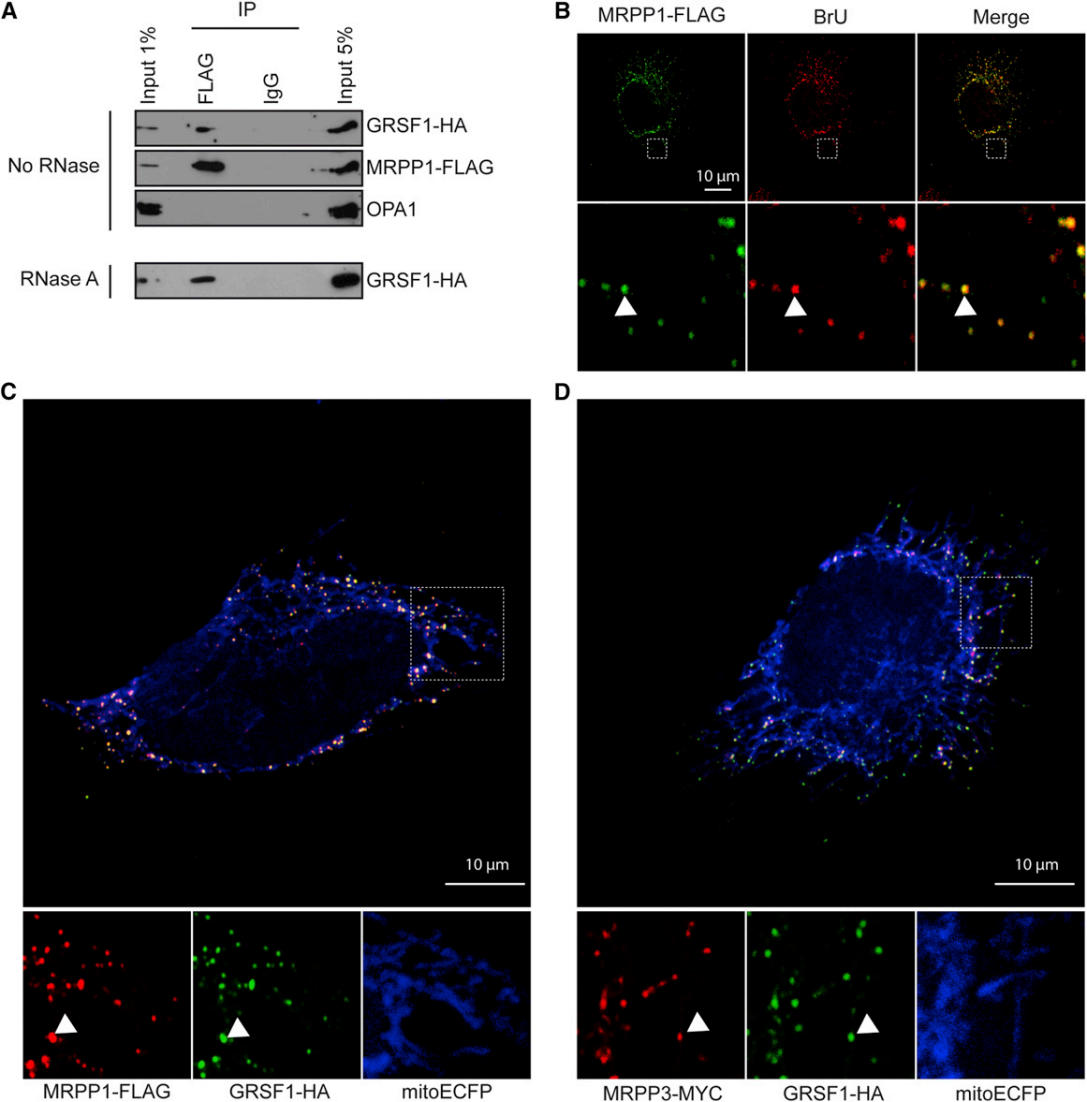
GRSF1 Interacts and Colocalizes with RNase P in Mitochondrial RNA Granules (A) Coimmunoprecipitation of MRPP1-FLAG and GRSF1-HA expressed in HEK293T cells. Pull-down was performed with FLAG antibodies or control IgG (IgG). OPA1 was used as a negative control. (B) Confocal analysis of HeLa cells transfected with MRPP1-FLAG, labeled with BrU, and immunolabeled with anti-FLAG and anti-BrU antibodies. Arrowheads, foci formed by BrU in mitochondria. Bottom panels, magnification. (C) Confocal analysis of HeLa cells transfected with MRPP1-FLAG, GRSF1-HA, and mitoECFP and immunolabeled with anti-FLAG and anti-HA antibodies. Arrowheads, foci formed by GRSF1 in mitochondria. Bottom panels, magnification. (D) Confocal analysis of HeLa cells transfected with MRPP3-MYC, GRSF1-HA, and mitoECFP and immunolabeled with anti-MYC and anti-HA antibodies. Arrowheads, foci formed by GRSF1 in mitochondria. Bottom panels, magnification.

To gain further insight into the mechanism by which GRSF1 regulates mtRNA levels, we searched for GRSF1 binding partners. For this experiment, GRSF1, along with any interacting proteins was affinity purified from HEK293T cells expressing GRSF1-FLAG, and the resulting protein mixture was analyzed by mass spectrometry (Table S1). Among the putative GRSF1 partners, RNase P showed the highest emPAI value (Ishihama et al., 2005), with a score of 35.9 and 8.2 for MRPP2 and MRPP1, respectively. To confirm the interaction between GRSF1 and MRPP1, we performed reverse coimmunoprecipitation in which HEK293T cells were transfected with MRPP1-FLAG and GRSF1-HA and immunoprecipitation was performed with anti-FLAG antibodies. We found that a significant amount of GRSF1-HA coimmunoprecipitated with MRPP1-FLAG, confirming our initial findings (Figure 4A). Similar results were obtained when RNA was degraded with RNase A prior to the pull-down assay, indicating that the interaction between GRSF1 and MRPP1 was not mediated by RNA. In agreement with these results, we observed that MRPP1 colocalized with GRSF1 and BrU in MRGs, with 92% of foci showing both MRPP1 and GRSF1 labeling (Figures 4B and 4C). MRPP3, the third component of RNase P, was not significantly enriched in the GRSF1 affinity purification. This was not unexpected, since the affinity between this subunit and the other two components is known to be weak and to be significantly decreased in the presence of salt (Holzmann et al., 2008). We therefore checked MRPP3 localization by immunocytochemistry using a MYC tag positioned at its C terminus. We found that, like MRPP1 and GRSF1, MRPP3 also accumulates in MRGs (Figure 4D). Finally, our results with RNase P prompted us to investigate whether RNase Z (ELAC2) also colocalizes with MRGs. However, in contrast to RNase P, ELAC2 was found to be diffuse in the mitochondrial matrix (Figure S3A).

In addition to RNase P, the coimmunoprecipitation experiment also identified a number of other putative GRSF1 partners, among which were several RNA-related proteins, including components of the cytosolic or mitochondrial ribosomes. This prompted us to test whether GRSF1 itself could be a component of mitoribosomes. However, using isokinetic sucrose density gradients we did not find cosedimentation of GRSF1 with either the mitoribosomal protein MRPL12 or DAP3 (Figure S3B).

Together, these data show that GRSF1 interacts and colocalizes with RNase P in mtRNA granules.

### Loss of GRSF1 Leads to Aberrant Mitochondrial RNA Processing

**Figure 5:**
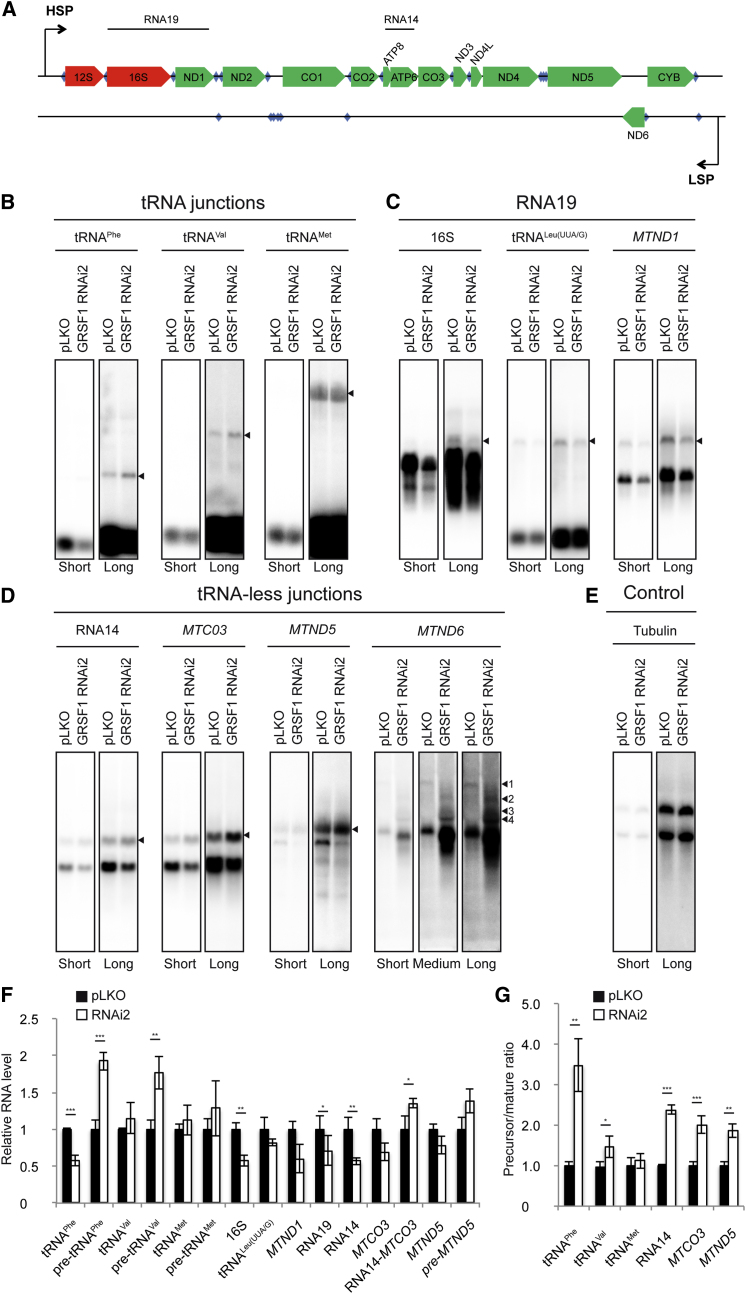
Loss of GRSF1 Leads to Aberrant Mitochondrial RNA Processing (A) Linear representation of the mitochondrial genome. Red, rRNA; green, ORF; blue, tRNA. RNA19 corresponds to the polycistronic 16S rRNA − tRNA^Leu(UUA/G)^ − *MTND1* RNA. RNA14 corresponds to bicistronic *MTATP8*-*MTATP6* RNA. HSP, heavy-strand promoter. LSP, light-strand promoter. (B) Northern blot analysis of tRNA-containing junctions from 143B cells treated with lentiviruses carrying an empty vector (pLKO) or GRSF1 RNAi2 using probes directed against mitochondrial tRNA^Ph*e*^, tRNA^Val^, and tRNA^Met^. Arrows indicate RNA precursors. Short and long correspond to different exposures. (C) Northern blot analysis of RNA19 (16S rRNA − tRNA^Leu(UUA/G)^ − *MTND1*) in 143B cells treated with lentiviruses carrying an empty vector (pLKO) or GRSF1 RNAi2 using probes directed against 16S rRNA, tRNA^Leu(UUA/G)^, or *MTND1*. Arrows indicate RNA19. Short and long correspond to different exposure time. (D) Northern blot analysis of tRNA-less RNA junctions in 143B cells treated with lentiviruses carrying an empty vector (pLKO) or GRSF1 RNAi2 using probes directed against RNA14 (*MTATP8* − *MTATP6*), *MTCO3*, *MTND5*, and *MTND6*. Arrows and numbers indicate RNA precursors. Short and long correspond to different exposure times. (E) Northern blot analysis of control from 143B cells treated with lentiviruses carrying an empty vector (pLKO) or GRSF1 RNAi2 using probes directed against tubulin. Note that two isoforms of tubulin are recognized by the probe. Short and long correspond to different exposure time. (F) Phosphorimager quantification of (B)–(D). *MTND6* was not quantified due to its diffused pattern. Data are normalized to tubulin and are shown as mean ± SEM (n ≥ 3). (G) Ratio between precursor and mature RNA, when applicable. *MTND6* was not quantified due to its diffused pattern. Data are shown as mean ± SEM (n ≥ 3).

RNase P has previously been reported to play a role in the processing of a number of transcripts that are flanked by tRNAs. RNase P cleaves tRNAs at their 5′ end and thereby participates in the so-called “tRNA punctuation model” (Ojala et al., 1981). This prompted us to test whether GRSF1 may cooperate with RNase P in the processing of mitochondrial primary transcripts involving tRNA excision (a linear representation of the mitochondrial genome can be found in Figure 5A). Using northern blot analyses of mtRNAs from control and GRSF1-depleted 143B cells, we observed a decrease in mature mitochondrially encoded tRNA^Phe^, 16S rRNA, tRNA^Leu(UUA/G)^, *MTND1*, RNA14, *MTCO3*, and *MTND5* in GRSF1-deficient cells, confirming the data obtained by Nanostring analysis as described above (Figure 3 and Figure 5). In contrast to the decrease in processed RNA, we found that several precursors, most notably tRNA^Phe^ and tRNA^Val^ precursors, were significantly increased in GRSF1-depleted cells (Figures 5B, 5F, and 5G). Similar results showing even more dramatic increases were obtained for tRNA-containing precursors in MRPP1-deficient cells (Figure S4) (Holzmann et al., 2008). However, in GRSF1-depleted cells, not all transcripts showed this expression profile, as, for example, steady-state levels of tRNA^Met^ remained unchanged (Figures 5B, 5F, and 5G).

Except for the 5′ upstream region of *MTCO1*, RNase P is not involved in the processing of precursor RNA devoid of flanking tRNA (Sanchez et al., 2011). We tested whether instead GRSF1 would play a more general role, including the processing of the tRNA-less RNA precursors. Analysis of RNA processing at the RNA14-*MTCO3*, *MTND5-CYB*, and *MTND6-*ncRNA junctions revealed a significant increase in the amounts of precursor transcripts for RNA14-*MTCO3* and *MTND5-CYB* (Figures 5D, 5F, and 5G). In addition, we found that *MTND6*, the only mitochondrial mRNA encoded by the light strand, was shorter in GRSF1-deficient cells (Figure 5D), whereas certain high-molecular-weight forms of *MTND6* accumulated in the absence of GRSF1 (Figure 5D, arrows 2, 3, and 4), while others decreased (Figure 5D, arrow 1). Thus, in contrast to RNase P, GRSF1 appears to be involved more generally in the processing of a wide range of primary transcripts.

Most mtRNA precursors are thought to have a short life span, although certain precursors have been reported to accumulate in mitochondria from human cells and tissue (Temperley et al., 2010). Among these, RNA19, a polycistronic RNA composed of 16S rRNA, tRNA^Leu(UUA/G)^, and *MTND1*, is one of the most abundant (Bindoff et al., 1993). The function of this stable RNA species is currently unknown, but its accumulation has been described in several human mitochondrial pathologies (Suzuki et al., 2011). Northern blot analysis of RNA19 using probes directed against 16S rRNA, tRNA^Leu(UUA/G)^, or *MTND1* showed a decrease in RNA19 in GRSF1 RNAi2-expressing cells (Figures 5C and 5F).

Our data indicate that mtRNA processing is strongly perturbed in the absence of GRSF1, in a manner that appears to extend beyond dysfunction of RNase P.

### Short-Term Transcription Inhibition Results in the Loss of Mitochondrial RNA Granules

**Figure 6:**
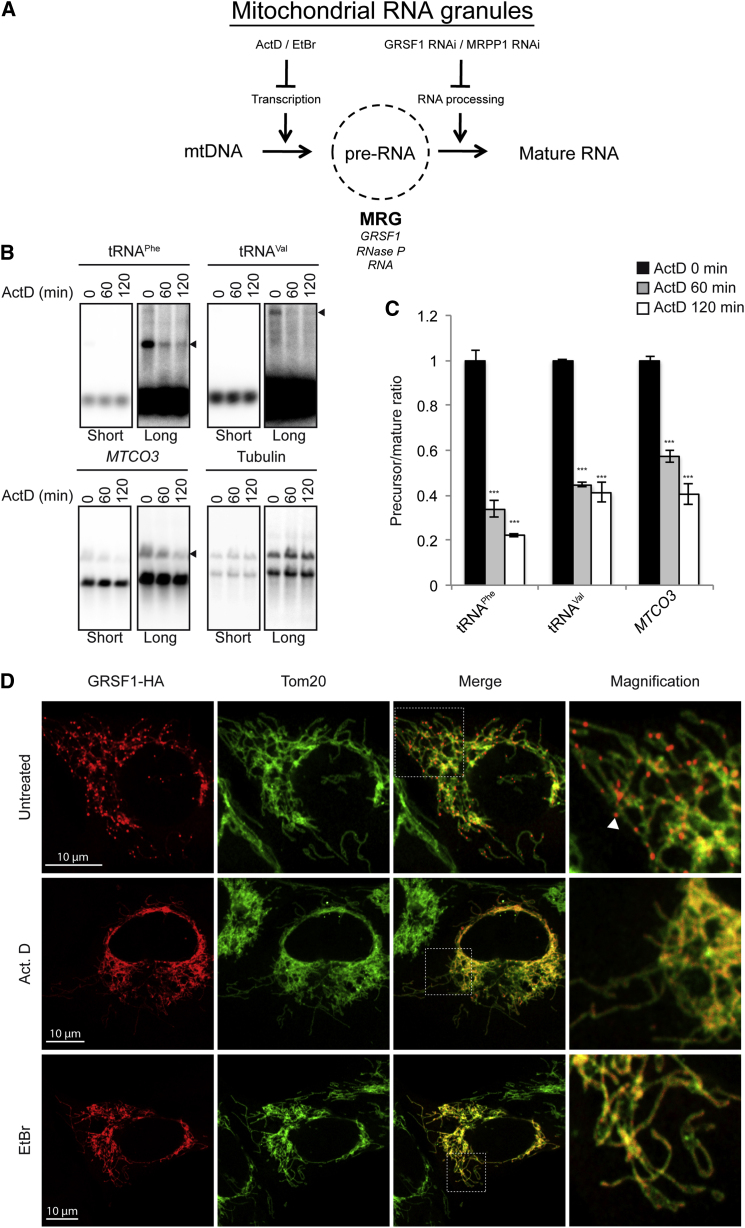
Transcription Inhibition Induces the Disappearance of Mitochondria RNA Granules (A) Working model: we hypothesize that nascent mitochondrially encoded precursor RNAs converge to mitochondrial RNA granules (MRGs). MRGs contain nascent RNAs, GRSF1, and RNase P. Inhibition of mitochondrial transcription with actinomycin D (ActD) or ethidium bromide (EtBr), as well as inhibition of RNA processing with RNAi against GRSF1 or MRPP1, is predicted to affect the formation of MRGs. mtDNA, mitochondrial DNA. (B) Northern blot analysis of 143B cells treated for 0, 60, or 120 min with 3 μM of ActD using probes directed against tRNA^Phe^, tRNA^Val^, *MTCO3*, and tubulin. Arrowheads, precursor RNA. Note that two isoforms of tubulin are recognized by the probe. Short and long correspond to different exposure times. (C) RNA precursor/mature ratio after phosphorimager quantification of (B). Data are shown as mean ± SEM (n = 3). (D) Confocal analysis of HeLa cells transfected with GRSF1-HA and treated for 120 min with 3 μM of ActD, 3 μg/ml EtBr. Immunolabeling was performed using anti-HA and anti-Tom20 antibodies. Arrowhead, MRG.

As mentioned above, MRGs are not observed in ρ^0^ cells that lack mtRNA (Figure S2). We hypothesized that RNA plays an essential role in the formation of MRGs. To test this hypothesis, we inhibited RNA transcription in 143B cells by treatment with 3 μM actinomycin D (Act D) for 2 hr (Figure 6A). Importantly, whereas transcription inhibition did not affect the level of mature tRNA^Phe^, tRNA^Val^, or *MTCO3* transcripts, it significantly affected their precursor RNAs (Figures 6B and 6C). We then tested whether transcription inhibition could have an impact on the formation of MRGs. HeLa cells transfected with GRSF1-HA were incubated for 2 hr with 3 μM ActD or 3 μg/mL ethidium bromide (EtBr), another transcription inhibitor, and were subsequently immunostained with anti-HA and anti-Tom20 antibodies. Whereas untreated cells showed MRGs, those treated with ActD or EtBr lacked MRGs as assessed by GRSF1 immunostaining. Indeed, in cells treated with transcription inhibitors, GRSF1 was homogenously distributed in the mitochondrial matrix (Figure 6D). Similar results were obtained using MRPP1-FLAG as a MRG marker (data not shown). All together, these experiments show a correlation between a reduction in the level of precursor RNAs and the disappearance of MRGs, suggesting that unprocessed transcripts may be required for the organization of MRGs.

### The Kinetics of RNA Release from MRGs Is Dependent on RNA Processing

**Figure 7:**
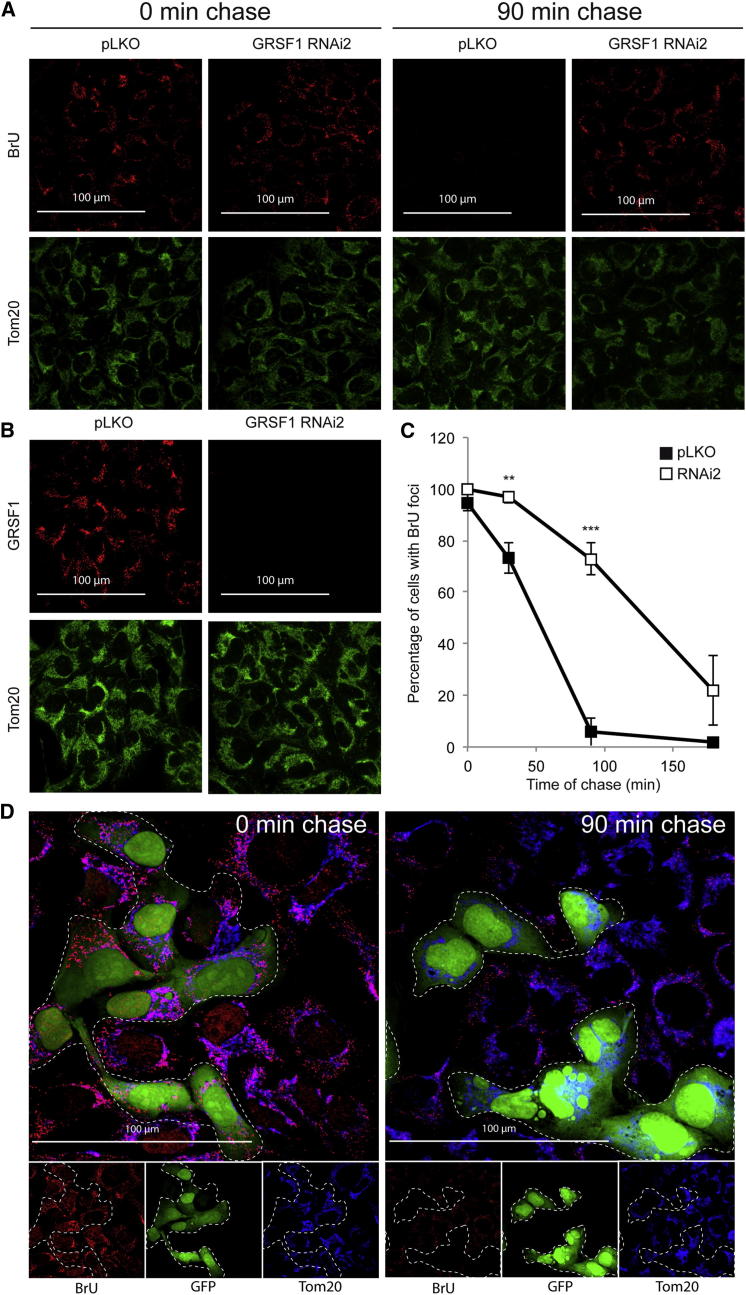
The Kinetics of RNA Release from MRGs Is Dependent on GRSF1 (A) Confocal analysis of 143B infected with pLKO or GRSF1 RNAi2-carrying lentiviruses after 60 min of BrU pulse and 0 or 90 min of chase with uridine. The parameters used for the entire analysis were identical for both conditions. Note that the thresholds were set up so that only the BrU foci could be visualized. (B) Confocal analysis of endogenous GRSF1 and Tom20 in pLKO or GRSF1 RNAi2-infected cells. The parameters used for the entire analysis were identical for both conditions. (C) Number of cells with BrU foci after 60 min of BrU pulse and up to 180 min of chase with uridine in 143B cells infected with pLKO or GRSF1 RNAi2-carrying lentiviruses. Cells were counted using widefield microscopy. Data are shown as mean ± SEM (n = 3). (D) Confocal analysis of BrU pulse chase of mixed control (green) and GRSF1 RNAi2 cells. Control 143B cells were infected with lentiviruses carrying an empty pLKO vector and lentiviruses carrying the GFP gene (pLKO + GFP), whereas GRSF1-depleted cells were infected with lentiviruses expressing GRSF1 RNAi2. All control cells were found to express GFP (data not shown). The two cell populations were mixed, selected with puromycin for 24 hr to eliminate those cells that would not have been infected by lentiviruses, and incubated with BrU for 60 min before chase with uridine. Cells were immunostained with anti-BrU and anti-Tom20 antibodies.

All data presented above suggest that MRGs may be sites where precursor RNA transcripts converge to be processed before release (Figure 6A). Based on this hypothesis, we predicted that altering RNA processing should increase the time RNA precursors would spend within the granules. To test this hypothesis, we measured the half-life of BrU-labeled MRGs following a BrU/uridine pulse chase. To perform this experiment, cells were labeled during a 60 min pulse of BrU followed by a chase with excess uridine for up to 180 min. As expected, before the chase, BrU-labeled transcripts were concentrated in MRGs (Figure 7A). Following addition of unlabeled uridine, the number of cells with mitochondrial BrU foci decreased with time up to 90 min, at which point no cell with labeled MRGs could be detected (Figures 7A and 7C). In agreement with previous results from Iborra and collaborators (Iborra et al., 2004), we estimated that the half-life of BrU foci was ∼50 min (Figure 7C). We next perturbed RNA processing by depleting cells of either GRSF1 or MRPP1, and then the half-life of BrU positive MRGs was measured. 143B cells were infected with lentiviruses carrying an empty vector or a shRNA against GRSF1, which led to a drastic reduction in GRSF1 expression in all cells (Figure 7B). BrU pulse-labeling experiments showed that the half-life of BrU-labeled MRGs was significantly longer in GRSF1-depleted cells (∼130 min) than in control cells (∼50 min) (Figures 7A and 7C). Similar results were obtained when GRSF1-depleted cells and control cells were mixed and cultured together for 24 hr, prior to BrU pulse-chase and microscopic analysis. Expression of GFP in the control cells allowed us to distinguish each population. Before chase, both control and GRSF1-deficient cells expressed BrU-labeled MRGs whose number and fluorescence intensity did not appear to differ between the two populations (Figure 7D). In contrast, after 90 min of chase, only the GRSF1-depleted cells showed labeled MRGs. A similar observation was made when RNA processing was altered by downregulation of MRPP1, where again we observed a strong delay in the disappearance of BrU-labeled MRGs (Figure S5A). This effect was unlikely to be the consequence of a defect in mitochondrial translation, since inhibition of translation using the protein synthesis inhibitor chloramphenicol did not modify the half-life of BrU-labeled MRGs (Figure S5B). All together, our data demonstrate that the presence of nascent RNA in MRGs is transient and that its release from MRGs depends on the efficiency of RNA processing.

## Discussion

Here, we report that isoform 1 of the RNA-binding protein GRSF1 colocalizes with nascent RNA and RNase P in a submitochondrial compartment that we have called “mitochondrial RNA granules” (MRGs). We found that GRSF1 is required for mtRNA expression and protein synthesis in mitochondria. Moreover, downregulation of GRSF1 or MRPP1, a subunit of RNase P, delays the transit of nascent RNA through MRGs, leading to reduced levels of mature mtRNA and reduced mitochondrial protein synthesis. These data strongly suggest that mtRNA processing and generation of functional RNA species are dependent on the integrity of the MRG.

Little is known about the mechanisms and regulation of mtRNA processing. We have identified GRSF1 as a key player in the processing of tRNA-containing and tRNA-lacking precursors. We have shown that GRSF1 binds the MRPP1 and MRPP2 subunits of RNase P, while the third subunit, MRPP3, was shown to colocalize with GRSF1 by immunohistochemistry. In addition, GRSF1 probably also acts in an RNase P-independent manner, since its activity is implicated in the processing of the tRNA-lacking precursors. Indeed, we found that GRSF1 is the first protein to be required for the processing of RNA14-*MTCO3* and *MTND6*-ncRNA precursors. The mechanism by which GRSF1 exerts these functions is still unclear, and it remains to be determined whether the protein is able to bind to specific nucleotide sequences present in these precursors.

Is GRSF1 also involved directly in mitochondrial protein synthesis, in addition to its role in RNA processing? We cannot rule this out, and the marked decrease in de novo mitochondrial protein synthesis upon GRSF1 depletion is perhaps more dramatic than would be predicted solely from the effects on RNA processing or stability. Moreover, we did see mitoribosomal proteins in the crude immunoprecipitation experiment. However, our sucrose gradient density fractionation showed no evidence of tight association between GRSF1 and mitoribosomal subunits. Further investigations will be required to understand whether GRSF1 could play a direct role in translation, whether a minor fraction of GRSF1 also interacts with the mature ribosomes, or whether the presence of mitoribosomal proteins in the immunoprecipitation corresponds to intermediates in the process of mitoribosome assembly.

We show that the MRGs are distinct from mitochondrial nucleoids. Only fewer than 10% of them overlapped with the DNA-containing structures, and these MRGs may correspond to BrU-labeled foci found in close proximity to nucleoids as described by Iborra et al. (2004). Nucleoids overlapping with newly synthesized RNA have been previously reported to contain the mitochondrial transcription factor TEFM and have therefore been described as transcriptionally active nucleoids (Minczuk et al., 2011). Taken together, these results suggest that the precursor transcripts may rapidly accumulate and may themselves promote the formation of MRGs by recruiting GRSF1, RNase P, and possibly other proteins involved in RNA precursor processing. This is supported by our findings that transient inhibition of transcription specifically affects the levels of precursor but not mature RNA and leads to the concomitant disappearance of MRGs. In contrast, inhibition of translation has no impact on the formation of MRGs, excluding the possibility that the disappearance of MRGs in cells exposed to transcription inhibitors is due to a drop in mitochondrial encoded proteins. How newly synthesized RNA dynamically induces the formation of granules is unknown, although this is likely to be through interaction with proteins, and thus a more complete analysis of the protein components of the MRG is likely to prove informative. In this context, it was recently proposed that RNA binding proteins containing low-complexity sequences could represent the organizing principle for the formation of cytosolic RNA granules (Han et al., 2012; Kato et al., 2012). Whether proteins of this type are involved in the formation of MRGs remains to be determined.

It is likely that MRGs are dynamic structures whose number and composition may vary according to the metabolic state of the cell. Based on the phenotypic consequences of GRSF1 and RNase P knockdown on RNA processing, we postulate that at least part of RNA processing occurs in MRGs. This does not exclude the possibility that GRSF1 and RNase P may have additional functions outside MRGs, nor does it exclude the possibility that MRGs may have other functions beyond a role in RNA processing. Based on our hypothesis on the role of MRGs in processing of tRNA-containing polycistronic transcripts, we expected that RNase Z, the enzyme that cleaves tRNA at its 3′ terminus, would be enriched in MRGs; however, this does not appear to be the case. Instead, RNase Z immunostaining is diffuse in the mitochondrial matrix, suggesting that the protein may have additional functions outside of MRGs and/or that its mitochondrial concentration is high enough in the matrix and not limiting for RNA processing in MRGs. Alternatively, RNase Z-induced cleavage of tRNA in precursor transcripts could occur outside of MRGs. Indeed, previous work has demonstrated that tRNAs are cleaved sequentially, initially at the 5′ end by RNase P, then at the 3′ end by RNase Z. Indeed, cells treated with RNAi against RNase P accumulate 3′ tRNA junctions (Sanchez et al., 2011). The MRG transcriptome analysis currently in progress in our laboratory should allow us to determine which precursor RNAs are found in these structures and may shed more light on the MRG-dependent events involved in mtRNA processing.

In previous experiments, Iborra et al. (2004) observed the formation of BrU-positive foci that decreased exponentially after chase. In agreement with their results, we calculated that the half-life of BrU-labeled RNA in MRGs is ∼50 min. The decrease in BrU-positive foci could be explained by RNA degradation or by its release into the matrix. Although we do not exclude the possibility of RNA degradation in MRGs, we favor the hypothesis that the granules are no longer visible when the transcripts have been released into the matrix, where their concentration drops significantly. Based on this hypothesis, we predicted that a dysfunction of the RNA processing machinery should lead to an increase in the time spent by nascent RNAs in MRGs. This prediction was confirmed by our observation that in the absence of GRSF1 or MRPP1, the persistence of BrU-positive MRGs was significantly increased compared to control.

In conclusion, we have identified a new mitochondrial subcompartment, the mitochondrial RNA granules, where GRSF1 and RNase P act together to ensure mtRNA processing. Further characterization of the proteome and transcriptome of these structures should prove to be very useful in advancing our understanding of mtRNA processing and may reveal other essential posttranscriptional steps involved in mitochondrial gene regulation.

## Experimental Procedures

### Cell Culture, Transfection, RNA Interference, and Chemicals

All cell culture reagents and chemicals were purchased from Sigma unless stated otherwise. Cells were cultured in Dulbecco’s modified Eagle medium (DMEM) supplemented with 10% heat-inactivated fetal bovine serum, 100 u/ml penicillin, 100 μg/ml streptomycin, and 2 mM L-glutamine in 5% CO_2_ at 37°C. 143B ρ^0^ cells were grown in the same medium supplemented with 110 μg/ml sodium pyruvate and 50 μg/ml uridine. Transfection of cells was done using Fugene (Roche). Mitochondrially targeted ECFP (mitoECFP) was from Clontech. Twinkle-EGFP was a gift from J.N. Spelbrink. MRPP1-FLAG and MRPP3-myc were gifts from W. Rossmanith. For RNA interference, 143B cells were transfected with interferin (Polyplus) or lipofectamine RNAiMax (Invitrogen) according to the the manufacturer’s instructions, and cells were analyzed 2–4 days posttransfection. pLKO.1 containing GRSF1 RNAi2 lentiviral vector was obtained from Sigma, and lentiviruses were produced according to the manufacturer’s instructions. After viral infection, cells were always selected for 24 hr with 3 μg/ml puromycin and left to recover for >24 hr before analysis. The same RNAi2 sequence was cloned into pLKO-TetON, a tetracycline-inducible version of pLKO (Addgene). Induction was achieved with 1 μg/ml tetracycline for 2–4 days. Inducible and noninducible versions of RNAi2 showed the same effect on RNA processing and were used indifferently to express RNAi2 (data not shown). RNAi sequences are described in Table S2.

### Bromouridine Staining, Immunofluorescence, and Microscopy

BrU pulse was performed using 5 mM 5-BrU (Sigma) for 60 min. For chase, cells were washed twice with culture medium and incubated in 25 mM uridine for the indicated times. Fixation was made in 4% paraformaldehyde for 10 min. Immunofluorescence was performed in PBS containing 3% BSA, 0.1% Triton X-100, and 20 u/ml RNasin (Promega). Microscopy was performed with ZEISS AXIOZ1 or ZEISS LSM700 confocal microscopes. To quantify the foci-foci colocalization, cells were labeled with antibodies against the protein/RNA of interest and GRSF1 and imaged, and >20 foci in >5 cells (>100 foci in total) per condition were blindly labeled on the protein/RNA pictures using ImageJ. Pictures were then merged, and the frequency of colocalization with GRSF1 foci was determined.

### Antibodies

Antibodies used were as followed: BrU/BrdU (Roche 11170376001), Tom20 (Santa Cruz FL-145), PHB (Neomarkers AB-1), OPA1 (BD PharMingen 612606), COX1 (Invitrogen 459600), COX2 (Invitrogen 35-8200), SDHB (Abcam AB14714), DAP3 (Abcam 13893), Actin (Sigma AC-40), and ELAC2 (Proteintech 10071-1-AP). GRSF1 antibodies were a gift from J. Wilusz, mtSSB from M. Zeviani, and HSP90 β from D. Picard. Cytochrome *c* antibodies were made in our laboratory. FITC, Texas red, or AMCA-coupled secondary antibodies were from Vector.

### RNA Extraction and Northern Blotting

Total RNA was extracted with TRI reagent (Sigma). RNA (15 μg) was separated on denaturing formaldehyde agarose gels and transferred to nylon membranes. Membranes were hybridized with ^32^P labeled in vitro-transcribed T7 RNA probes in 50% formamide, 7% SDS, 0.2 M NaCl, 80 mM sodium phosphate (pH 7.4), and 100 μg/ml salmon sperm DNA. Hybridization was carried out at 60°C for 3 hr. Membranes were subsequently rinsed in 1× SSC, 0.1% SDS and washed for 30 min in 0.5% SSC, 0.1% SDS. Imaging and quantification were performed with a phosphorimager (Bio-Rad). Probes are described in Table S2.

### NanoString

The NanoString analysis was performed by the genomics platform of the University of Geneva. HDAC, NUP155, and tubulin were used to normalize the data. Probes are described in Table S2.

### Mitochondria-Rich Fraction Isolation, Proteinase K Accessibility Test, and Alkali Treatment

Cell fractionation was done in MB buffer (210 mM mannitol, 70 mM sucrose, 10 mM HEPES-KOH [pH 7.4], 1 mM EDTA) using a glass homogenizer (Kontes). Nuclei and unbroken cells were discarded after centrifugation for 5 min at 2,000 g. The mitochondria-rich fraction corresponded to the pellet obtained after a 10 min centrifugation at 13,000 g. For the PK accessibility test, mitochondria were resuspended at a final concentration of 0.5 μg/μl in MB, swelling buffer (10 mM HEPES-KOH [pH 7.4]), or MB + 0.2% (v/v) Triton X-100. PK was added to a final concentration of 50 μg/ml, and the mixture was incubated on ice for 20 min. PMSF (2 mM) was then added to inhibit the PK. After TCA precipitation, samples were analyzed by western blotting.

For alkaline sodium carbonate extraction, 100 μg of mitochondria was resuspended in 100 mM Na_2_CO_3_ (pH 11.5) and incubated on ice for 30 min before a 30 min centrifugation at 100,000 g.

### Pulse Labeling of Mitochondrial Translation

143B cells were incubated for 20 min in methionine/cysteine-free DMEM (Sigma) complemented with dialysed serum and 2 mM L-glutamine. Cells were then incubated for 1 hr in the same medium in the presence of 100 μg/ml emetine and 100 μCi/ μL ^35^S-labeled methionine/cysteine (PerkinElmer). Total protein concentration of cell lysates was measured by Bradford (Bio-Rad), and lysates were resolved on a 15%–20% tricine gel, transferred to a nitrocellulose membrane, and analyzed by autoradiography.

### Import of Recombinant Radiolabeled GRSF1 into Isolated Mitochondria

In vitro synthesis of radiolabeled GRSF1 isoforms was performed with TNT kit (Promega). Import into isolated mitochondria was performed according to Koppen et al. (2009). PK was used instead of trypsin to degrade nonimported material.

### Affinity Purification of GRSF1-Interacting Proteins and Coimmunoprecipitation

Affinity purification and identification of GRSF1-interacting proteins were performed on mitoplasts from GRSF1-FLAG-expressing HEK293T cells as previously described (Richter et al., 2010). To confirm the interaction between MRPP1 and GRSF1, HEK293T cells were transfected with MRPP1-FLAG and GRSF1-HA. Two days after transfection, mitochondria were isolated and lysed in lysis buffer (50 mM Tris-HCl [pH 7.4], 150 mM NaCl, 10 mM MgCl_2_, 1 mM EDTA, 1% [v/v] Triton X-100, and protease inhibitor cocktail [Roche]). Coimmunoprecipitation of MRPP1-FLAG with GRSF1-HA was performed on mitochondria from HEK293T cells expressing both constructs. Mitochondria were homogenized in lysis buffer, and pull-down was performed using polyclonal anti-FLAG antibodies (Sigma). After washing and elution in SDS-containing loading buffer, proteins were separated by SDS-PAGE. RNase A treatment was performed by adding 100 μg/ml RNase A (Fermentas) during lysis.

### Isokinetic Sucrose Gradient

143B cells were lysed in lysis buffer and layered on a 2 ml 10%–30% (w/v) sucrose gradient as previously described (Richter et al., 2010). Samples were centrifuged for 3 hr at 100,000 g; 100 μl fractions were collected and separated by SDS-PAGE.

### Statistical Analysis

Data are represented as mean ± SEM and are representative of more than three independent experiments. Stars correspond to a two-tailed Student t test, with ^∗^p < 0.05, ^∗∗^p < 0.01, and ^∗∗∗^p < 0.001.

## Supplemental Information

Document S1. Figure S1, Figure S2, Figure S3, Figure S4, Figure S5, and Table S1

Table S2. Related to the Experimental Procedures
