# Supplementary material for: GRSF1 Regulates RNA Processing in Mitochondrial RNA Granules
Source: Cell Metab. 2013 Mar 5;17(3):399–410. doi: 10.1016/j.cmet.2013.02.005 (PMC3593211; doi:10.1016/j.cmet.2013.02.005)
Supplement: Document S1. Figure S1, Figure S2, Figure S3, Figure S4, Figure S5, and Table S1 [file mmc1.pdf]

Cell Metabolism, Volume *17*

Supplemental Information

GRSF1 Regulates RNA Processing in Mitochondrial RNA Granules

Alexis A. Jourdain, Mirko Koppen, Mateusz Wydro, Chris D. Rodley, Robert N. Lightowlers, Zofia M. Chrzanowska-Lightowlers, and Jean-Claude Martinou

Inventory of Supplemental Information

- Figure S1 : Analysis of GRSF1 isoforms in different cell lines. Related to Figure 1.
- Figure S2 : Analysis of GRSF1 in 143B ρ^0 cells. Related to Figure 2.
- Figure S3 : GRSF1 does not co-localize with RNase Z and is not associated with mitochondrial ribosomes. Related to Figure 4.
- Figure S4 : MRPP1 knock-down. Related to Figure 5.
- Figure S5 : Effect of MRPP1 RNAi and translation inhibition on BrU pulse-chase. Related to Figure 7.
- Table S1 : Putative GRSF1-interacting proteins. Related to Figure 4.
- Table S2 : Related to the Experimental Procedures.

Supplemental Figures

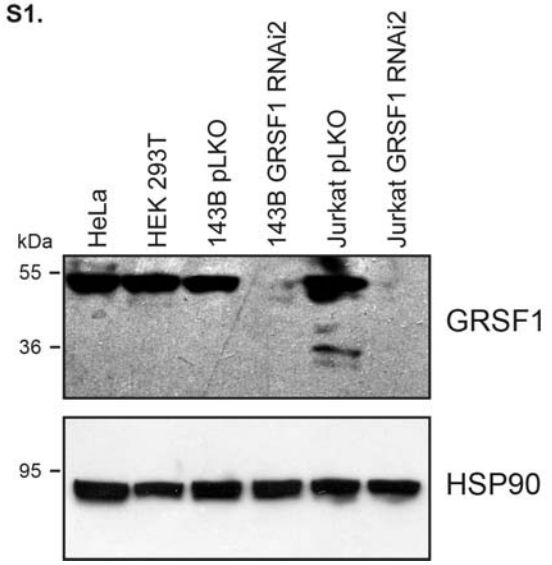

Figure S1 : Analysis of GRSF1 isoforms in different cell lines. Immunoblot analysis of GRSF1 isoforms in HeLa, HEK 293T, 143B and Jurkat cells. Cells were infected with lentiviruses carrying an empty vector (pLKO) or a shRNA against GRSF1 (RNAi2). No low molecular weight form of GRSF1 was observed in HeLa, HEK 293T or 143B cells, even after a long exposure time.

S2.

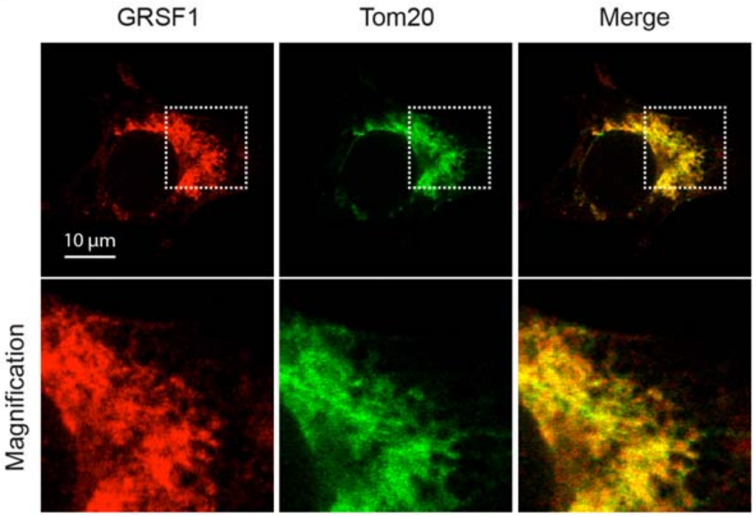

Figure S2 : Analysis of GRSF1 in 143B ρ^0 cells. 143B ρ^0 cells were immunolabeled with antibodies against GRSF1 and Tom20.

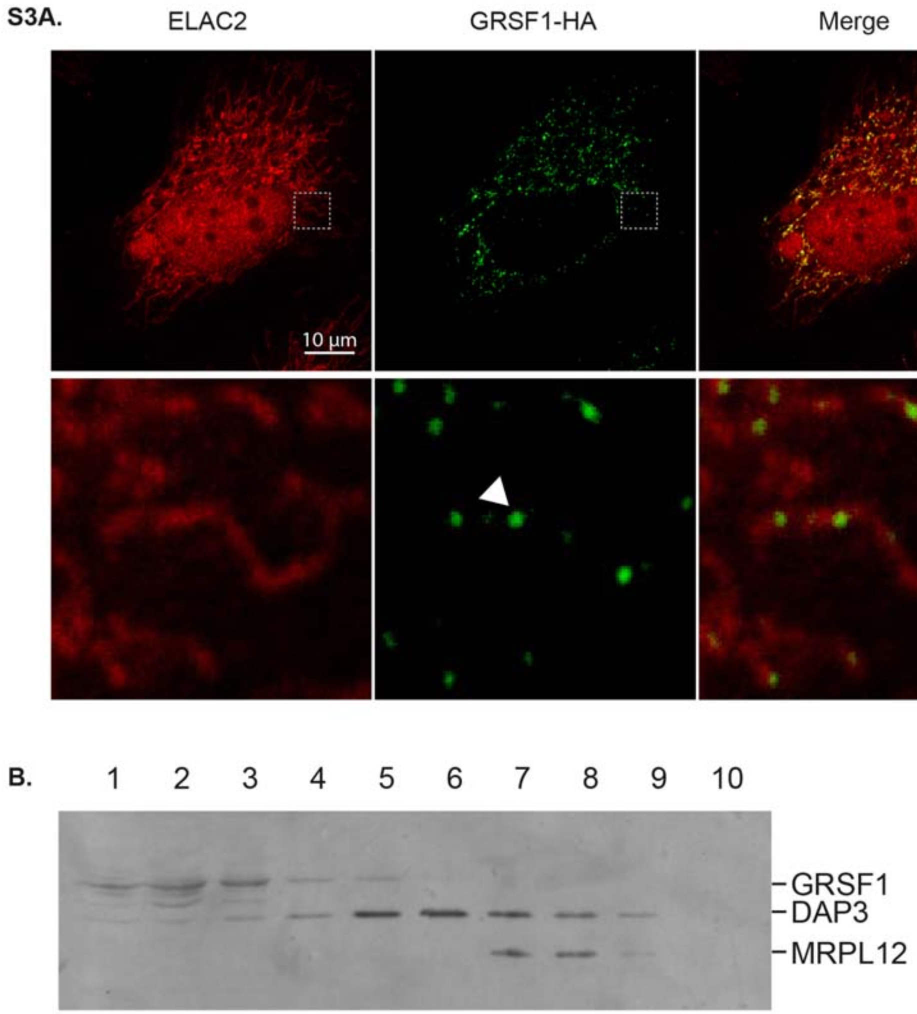

Figure S3 : GRSF1 does not co-localize with RNase Z and is not associated with mitochondrial ribosomes. A. Immunolabeling of HeLa cells transfected with GRSF1-HA using anti-HA and anti-ELAC2 antibodies. Arrowhead : mitochondrial foci corresponding to GRSF1. B. Immunoblot analysis of HeLa cell extracts after isokinetic sucrose gradient separation. MRPL12 is a protein of the large mitoribosomal subunit. DAP3 is a protein of the small mitoribosomal subunit. 1: top fraction (low molecular weight), 10: bottom fraction (high molecular weight).

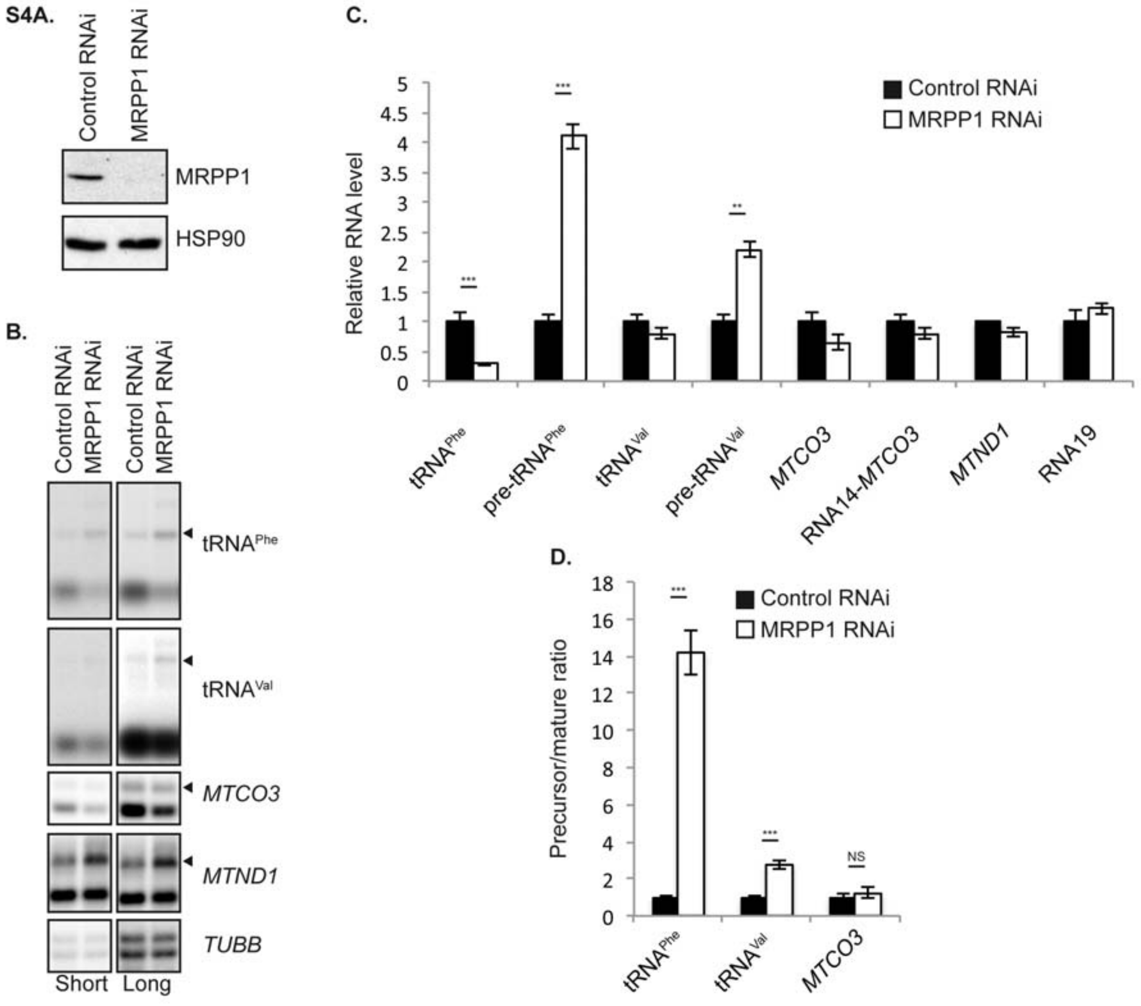

Figure S4 : MRPP1 knock-down. A. Immunoblot analysis of cell lysates prepared from cells transfected with a control RNAi or an RNAi directed against MRPP1. HSP90 was used as a loading control. B. Northern blot analysis of 143B cells treated with control or MRPP1 RNAi using probes for various RNA. Arrowheads indicate RNA precursors. *MTND1* precursor corresponds to RNA19. Short and long correspond to different exposure time. C. Phosphorimager quantification. Data are normalized to tubulin and are shown as mean ± SEM (N ≥ 3). D. Ratio between precursor and mature RNA, when applicable. Data are shown as mean ± SEM (N ≥ 3).

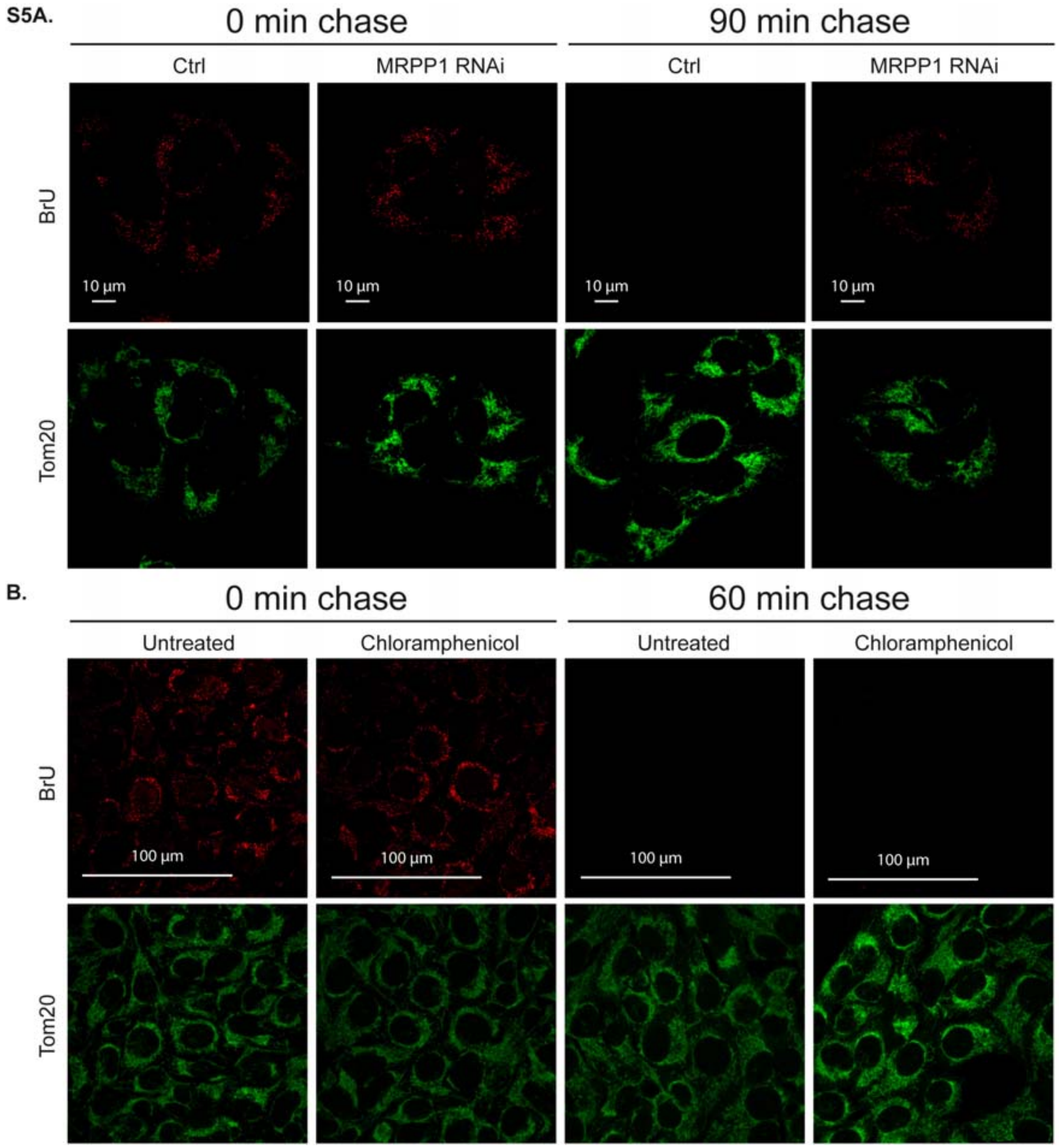

Figure S5 : Effect of MRPP1 RNAi and translation inhibition on BrU pulse-chase. A. Confocal analysis of 143B treated with control or MRPP1 RNAi after 60 min of BrU pulse and before (0) or after 90 minutes of chase with uridine. The parameters used for the entire analysis were identical for both conditions. B. Confocal analysis of 143B cells cultured in the absence or presence of 20 µg/ml chloramphenicol. Chloramphenicol was maintained during the pulse and the chase. BrU/uridine pulse chase and imaging was monitored as in (A).

Table S1

Rank	Gene	UNIPROT	PROT ID	PROT NAME	emPAI value Control IP 1	GRSF1 IP	Control IP 2
1	HSD17B10	Q99714	NP_004484.1	hydroxysteroid (17-beta) dehydrogenase 10 isoform 1 (RNaseP MRPP2)	0.71	35.87	0.71
2	GRSF1	Q12849-2	NP_002083.2	G-rich RNA sequence binding factor 1	0.00	26.83	0.53
3	RG9MTD1	Q9NRG5	NP_060289.2	RNA (guanine-9-) methyltransferase domain containing 1 (RNaseP MRPP1)	0.05	8.15	0.00
4	MRPS26	Q9BYN8	NP_110438.1	mitochondrial ribosomal protein S26	1.25	6.63	0.31
5	MRPL11	Q9Y3B7	NP_057134.1	mitochondrial ribosomal protein L11 isoform a	0.25	6.20	0.00
6	TFAM	Q00059	NP_003192.1	transcription factor A, mitochondrial	0.30	6.02	0.43
7	NME4	O00746	NP_005000.1	nucleoside-diphosphate kinase 4	0.00	5.45	0.00
8	MRPS25	P82663	NP_071942.1	mitochondrial ribosomal protein S25	1.00	5.31	0.12
9	HSPA9	P38646	NP_004125.3	heat shock 70kDa protein 9B precursor	0.96	4.10	0.80
10	RPL28	P46779	NP_000982.2	ribosomal protein L28	0.00	4.01	0.00
11	RPL14	P50914	NP_001030168.1	ribosomal protein L14	0.00	3.22	0.00
12	MRPS34	P82930	NP_076425.1	mitochondrial ribosomal protein S34	0.10	3.22	0.10
13	MRPS22	P82650	NP_064576.1	mitochondrial ribosomal protein S22	0.92	3.06	0.35
14	MRPL47	Q9HD33	NP_065142.2	mitochondrial ribosomal protein L47 isoform a	0.23	2.90	0.00
15	RNMTL1	Q9HC36	NP_060616.1	RNA methyltransferase like 1	0.00	2.87	0.00
16	MRPL48	Q96GC5	NP_057139.1	mitochondrial ribosomal protein L48	0.47	2.59	0.29
17	MRPS7	Q9Y2R9	NP_057055.1	mitochondrial ribosomal protein S7	0.53	2.59	0.19
18	ATP5B	P06576	NP_001677.2	ATP synthase, H+ transporting, mitochondrial F1 complex, beta subunit precursor	0.87	2.51	1.39
19	ATP5O	P48047	NP_001688.1	mitochondrial ATP synthase, O subunit precursor	0.78	2.16	0.00
20	MRPS31		NP_005821.1	mitochondrial ribosomal protein S31	0.33	1.99	0.19
21	ACOT9		NP_001028755.2	acyl-Coenzyme A thioesterase 2, mitochondrial isoform b	0.00	1.98	0.08
22	HADHA	P40939	NP_000173.2	mitochondrial trifunctional protein, alpha subunit precursor	0.16	1.97	0.19
23	C1QBP	Q07021	NP_001203.1	complement component 1, q subcomponent binding protein precursor	0.19	1.89	1.22
24	HARS2	P49590	NP_036340.1	histidyl-tRNA synthetase-like	0.00	1.87	0.00
25	MRPS15	P82914	NP_112570.2	mitochondrial ribosomal protein S15	0.33	1.61	0.21
26	MRPS2	Q9Y399	NP_057118.1	mitochondrial ribosomal protein S2	0.00	1.55	0.33
27	DHX30	Q7L2E3-2	NP_619519.1	DEAH (Asp-Glu-Ala-His) box polypeptide 30 isoform 3	0.00	1.54	0.10
28	PTCD3	Q96EY7	NP_060422.4	Pentatricopeptide repeat domain 3	0.25	1.53	0.25
29	MRPL24	Q96A35	NP_663781.1	mitochondrial ribosomal protein L24	0.00	1.47	0.00
30	MRPS27	Q92552	NP_055899.1	mitochondrial ribosomal protein S27	0.36	1.32	0.30
31	PAPD1	Q9HA74	NP_060579.2	PAP associated domain containing 1	0.00	1.23	0.00
32	MRPL2	Q5T653	NP_057034.2	mitochondrial ribosomal protein L2	0.00	1.22	0.19
33	MRPL21	A6NKU0	NP_852615.1	mitochondrial ribosomal protein L21 isoform d	0.09	1.22	0.00
34	DAP3	P51398	NP_387506.1	death-associated protein 3	0.39	1.12	0.26
35	MRPL4	Q9BYD3	NP_057040.2	mitochondrial ribosomal protein L4 isoform a	0.31	1.11	0.07
36	PNPT1	Q8TCS8	NP_149100.1	polyribonucleotide nucleotidyltransferase 1	0.08	1.04	0.03
37	MRPL28	Q13084	NP_006419.2	mitochondrial ribosomal protein L28	0.00	1.03	0.00
38	MDH2	P40926	NP_005909.2	mitochondrial malate dehydrogenase precursor	0.43	1.03	0.19
39	MRPS28	Q9Y2Q9	NP_054737.1	mitochondrial ribosomal protein S28	0.49	1.02	0.22

Table S1: Putative GRSF1-interacting proteins. Ranked list of the proteins co-immunoprecipitating with GRSF1-FLAG, according to their emPAI value.
